# Terminal differentiation of T cells is strongly associated with CMV infection and increased in HIV-positive individuals on ART and lifestyle matched controls

**DOI:** 10.1371/journal.pone.0183357

**Published:** 2017-08-14

**Authors:** Thijs Booiman, Ferdinand W. Wit, Arginell F. Girigorie, Irma Maurer, Davide De Francesco, Caroline A. Sabin, Agnes M. Harskamp, Maria Prins, Claudio Franceschi, Steven G. Deeks, Alan Winston, Peter Reiss, Neeltje A. Kootstra, on behalf of The Co-morBidity in Relation to Aids (COBRA) Collaboration

**Affiliations:** 1 Department of Experimental Immunology, Amsterdam Infection and Immunity Institute, Academic Medical Center, University of Amsterdam, Amsterdam, The Netherlands; 2 Amsterdam Institute for Global Health and Development, Amsterdam, The Netherlands; 3 Department of Global Health & Division of Infectious Disease, Academic Medical Center, University of Amsterdam, Amsterdam, The Netherlands; 4 HIV Monitoring Foundation, Amsterdam, The Netherlands; 5 Department of Infection and Population Health, University College London, London, United Kingdom; 6 Public health service, Amsterdam, The Netherlands; 7 Department of Experimental, Diagnostic and Specialty Medicine, Alma Mater Studiorum Universita di Bologna, Bologna, Italy; 8 Department of Medicine, University of California, San Francisco, California, United States of America; 9 Imperial College of Science, Technology and Medicine, London, United Kingdom; Universita Vita Salute San Raffaele, ITALY

## Abstract

HIV-1-positive individuals on successful antiretroviral therapy (ART) are reported to have higher rates of age-associated non-communicable comorbidities (AANCCs). HIV-associated immune dysfunction has been suggested to contribute to increased AANCC risk. Here we performed a cross-sectional immune phenotype analysis of T cells in ART-treated HIV-1-positive individuals with undetectable vireamia (HIV-positives) and HIV-1-negative individuals (HIV-negatives) over 45 years of age. In addition, two control groups were studied: HIV negative adults selected based on lifestyle and demographic factors (Co-morBidity in Relation to AIDS, or COBRA) and unselected age-matched donors from a blood bank. Despite long-term ART (median of 12.2 years), HIV-infected adults had lower CD4^+^ T-cell counts and higher CD8^+^ T-cell counts compared to well-matched HIV-negative COBRA participants. The proportion of CD38^+^HLA-DR^+^ and PD-1^+^ CD4^+^ T-cells was higher in HIV-positive cohort compared to the two HIV-negative cohorts. The proportion CD57^+^ and CD27^−^CD28^−^ cells of both CD4^+^ and CD8^+^ T-cells in HIV-positives was higher compared to unselected adults (blood bank) as reported before but this difference was not apparent in comparison with well-matched HIV-negative COBRA participants. Multiple regression analysis showed that the presence of an increased proportion of terminally differentiated T cells was strongly associated with CMV infection. Compared to appropriately selected HIV-negative controls, HIV-positive individuals on ART with long-term suppressed viraemia exhibited incomplete immune recovery and increased immune activation/exhaustion. CMV infection rather than treated HIV infection appears to have more consistent effects on measures of terminal differentiation of T cells.

## Introduction

Antiretroviral therapy (ART) for human immunodeficiency virus type 1 (HIV-1) infection has dramatically reduced AIDS-associated morbidity and mortality [1–3]. However, HIV-1-positive individuals on successful ART are reported to have higher rates of age-associated noncommunicable comorbidities (AANCCs) than the general population [3–7]. Several contributing factors have been implicated, including ART toxicity, chronic immune activation, immune dysfunction and a higher prevalence of traditional risk factors [8, 9]. Interestingly, immunological alterations observed during treated HIV-1 infection reflect those observed in the general population during aging [7, 10–12]. These include high levels of soluble inflammatory and coagulation related proteins, high levels of T cell activation, high levels of T cell exhaustion, low levels of naïve T cells and an extensive proliferative history of CD8 T cells [13–18]. Together, these age-associated immunological alterations are referred to as immune senescence [19, 20], although the precise definition and clinical significance of this syndrome remains controversial [4, 21, 22].

The immune senescent phenotype was first reported in a group of elderly adults who progressed more rapidly [23]. Subsequent work suggested that chronic viral infections such as cytomegalovirus (CMV), hepatitis B virus (HBV), hepatitis C virus (HCV) and HIV, contribute to the development of this phenotype [8, 13, 15, 24–27]. Indeed, CMV infection is also associated with low CD4:CD8 ratios, increased systemic inflammation and increased expansion of terminally differentiated and senescent T cells [13, 15, 28, 29]. CMV prevalence is extremely high in HIV-positive individuals and therefore CMV infection may well contribute to the immune senescent phenotype observed in HIV-positive individuals. Recently, we demonstrated that levels of terminal differentiation of T cells and immune senescence did not differ between HIV-positive individuals on ART and HIV-negative controls with comparable age, lifestyle and demographic characteristics [30], findings which contrasted with those from other studies [13–16]. To increase our understanding of these findings, we analysed the effect of HIV-1 and CMV infection on T cell activation, exhaustion and terminal differentiation of T cells in HIV-1-positive individuals with suppressed viraemia on ART (HIV-positive), HIV-1-negative individuals (HIV-negative) comparable regarding most lifestyle and demographic factors derived from the Co-morBidity in Relation to AIDS (COBRA) cohort, and age-matched unselected blood bank donors (referred in the tables as BBD). Notably, these blood bank donors are at lower risk of acquiring blood borne infections than the general population but often used as a control group for comparative studies.

## Materials and methods

### Subjects

Study subjects other than the bloodbank donors participated in an ongoing European Commission-funded project known as COBRA (Co-morBidity in Relation to AIDS). COBRA is a detailed, prospective evaluation of the burden of AANCC among 134 HIV-1-positive patients on ART and 79 appropriately chosen non-infected controls who have comparable socio-demographic and behavioral (risk) factors. COBRA aims to provide a robust estimate of the effect of treated HIV infection on the prevalence, incidence and age of onset of AANCC. Furthermore, COBRA aims to clarify the pathogenic mechanisms underlying this causative link, including the possible induction of an inflammation-associated accelerated ageing phenotype. Exclusion criteria were: age under 45 years, and self-reported current intravenous drug use (in the past six months), daily use of recreational drugs (with the exception of cannabis), and excess alcohol intake (>48 units per week). All HIV-positive participants were required to be on ART and to have had undetectable plasma HIV RNA (<50 copies/mL) for ≥12 months prior to enrolment. Participants were recruited from two study sites: London and Amsterdam. For this immunological study, 40 HIV-positive and 40 HIV-negative participants were randomly selected with equal numbers in each of the following age-groups (45–50, 51–55, 56–60, 61–65, 66–70, and 70+), except for the oldest age category in which only a few individuals were available. Materials from blood bank donors were obtained from the Dutch national blood bank in Amsterdam, the Netherlands (www.sanquin.nl). Blood bank donors (median: 58, IQR: 52.0–65.0) were matched for age with the HIV-positive (Median: 58.5, IQR: 53.5–63.5) and HIV-negative (median: 59.0, IQR: 53.0–64.5) COBRA participants and selected in such a way that the different age categories in the COBRA, except for the category of 70+, were equally represented. Blood bank donors from the Netherlands are actively screened for HIV, Hepatitis B, Hepatitis C, syphilis, and HTLV infection. Individuals aged above 70 years or individuals that display high risk behavior for blood born infections are excluded from blood donation. Of note, blood bank donors infected with HBV but who cleared the infection documented by an anti-HBs antibody titer of at least 200 IU/L or blood bank donors that have been vaccinated against HBV are not excluded from blood donation.

### Ethics statement

This study has been conducted in accordance with the ethical principles set out in the declaration of Helsinki and was approved by the institutional review board of the Academic Medical Center (AMC) (NL 30802.018.09), the London (Stanmore) Research Ethics Committee (REC) (13/LO/0584), and the Ethics Advisory Body of the Sanquin Blood Supply Foundation in Amsterdam. Written informed consent was obtained from all participants.

### T cell phenotyping and flow cytometry

Cryopreserved PBMC were thawed and cell viability was analysed by trypan blue staining and for FACS analysis cell viability was required to be >75%. PBMC were stained with monoclonal antibodies for 30min at 4°C in the dark, to determine expression levels of different T-cell surface molecules. T cell differentiation was defined as the proportion of naïve (CD45RA^+^CD27^+^CCR7^+^), central memory (CD45RA^−^CCR7^+^CD27^+^), transitional memory (CD45RA^−^CCR7^−^CD27^+^), effector memory (CD45RA^−^CCR7^−^CD27^−^), and terminally differentiated effector memory (TEMRA; CD45RA^+^CCR7^−^CD27^−^) within the total CD4 or CD8 T cell population. T cell activation was defined as the proportion of cells that were positive for both CD38 and HLA-DR within the total CD4 or CD8 T cell population. T cell exhaustion was defined as the proportion of PD1 positive cells within the total CD4 or CD8 T cell population. Terminally differentiated T cells were defined as proportion of CD57 positive cells within the total CD4 or CD8 T cells population, the proportion of cells negative for both CD27 and CD28 within the total CD4 or CD8 T-cells population, or the proportion of CD57 positive within the CD28^−^CD4^+^ or CD28^−^CD8^+^ T cell populations. The following directly conjugated monoclonal antibodies were used for cell surface marker staining: CD3 V500, CD4 PE-Cy7, HLA-DR Fitc, CD38 PE, CD28 PerCP Cy5.5, CD45RA PE-Cy7, CD8 Pacific Blue, CD57 APC, CCR7 PE, CD27 PerCP Cy5.5 (BD Biosiences, San Jose, CA, USA), CD27 APCeFluor780, CD4 APC eFluor780, and PD-1 PE (eBioscience, San Diego, CA, USA). Fluorescence was measured with the FACS Canto II (BD Biosciences). The proportion of cells expressing each marker were determined using FlowJo 7.6 (TreeStar, Ashland, OR, USA). The gating strategy is displayed in the supplementary information (S1 Fig).

### CMV antibody titers

CMV specific IgG levels are believed to be a surrogate marker of CMV reactivation and concurrent immune response to control infection [31]. Thus, increased CMV total antibody and high avidity titers indicate recurrent CMV antigen exposure, whereas CMV high avidity titers are also indicative of increased antibody maturation. CMV total antibody titers and high avidity antibody titers were measured by ELISA-VIDITEST anti-CMV-IgG and IgG avidity (VIDIA, Praha, Czech republic) according to the manufacturer’s instruction. For quantification a standard curve was prepared by serial dilution of plasma from a known CMV seropositive individual.

### Statistical analysis

Differences in subject characteristics between HIV-positive, HIV-negative and blood bank donors were assessed using Student’s T test, Mann-Whitney U test, or Pearson Chi-square test, as appropriate. Associations of immunological markers with study group (HIV-positive, HIV-negative, blood bank donors) and CMV serostatus were evaluated using multivariable linear regression adjusted for age and gender. In the sub-group of participants who were CMV seropositive, we then considered the association of each immunological marker with group and i) CMV total IgG and ii) CMV high avidity IgG, with the same adjustments. Some outcomes were transformed to attain normality as indicated in the table. Unstandardized coefficient (B) indicates the difference in the outcome (immunological markers) with respect to the variables (study group, CMV infection), and are given in the table. Uncorrected p values are given in the tables and the required p value for significance after Bonferroni adjustment for multiple testing is indicated. Analyses were performed in IBM SPSS Statistics for Windows v.23 (IBM, Armonk, NY, USA) and GraphPad Prims 6 (GraphPad, La Jolla, CA, USA).

## Results

### Baseline characteristics of COBRA participants and blood bank donors

HIV-positive individuals had a median (IQR) age of 58.5 (53.5–63.5) years and HIV-negative controls had a median (IQR) age of 59 (53–63.5) years. The groups were representative of the main COBRA cohort study (HIV-positive individuals: 93.3% male, 86.6% MSM, 12.0% of African descent; HIV-negative controls: 92.4% male, 79.8% MSM, 2.6% of African descent). HIV-positive individuals had been diagnosed with HIV for a mean (standard deviation) of 13.9 (4.8) years ago, had been on ART for a mean (standard deviation) of 12.2 (4.7) years and had spent a median (IQR) of 8.0 (5.3–10.9) years with an undetectable plasma viral load (Table 1). Whilst the percentage of males did not differ significantly between the HIV-positive and HIV-negative COBRA participants, it was significantly lower in the group of blood bank donors. HIV-positive and HIV-negative COBRA participants were more often (co-)infected with CMV and HCV as compared to the blood bank donors, whereas HIV-positive COBRA participants were more often infected with CMV, HBV and HCV when compared to HIV-negative COBRA participants (Table 1). HIV-positive COBRA participants, despite long-term suppression of HIV-replication by ART, exhibited incomplete CD4^+^ T-cell restoration and elevated CD8^+^ T-cell counts compared to HIV-negative COBRA participants, as reflected by lower CD4 counts, higher CD8 counts and an inverted CD4:CD8 T-cell ratio (Table 1).

**Table 1:** Baseline characteristics HIV-positive and HIV-negative COBRA participants and blood bank donors.

	HIV-positive n = 40	HIV-negative n = 40	HIV-positive vs HIV-negative	Blood bank donors n = 35	HIV-positive vs BBD	HIV-negative vs BBD
	n (%) or median (IQR)	n (%) or median (IQR)	*p* value^1^	n (%) or median (IQR)	*p* value^1^	*p* value^1^
Age (Years)	58.5 (53.5–63.5)	59.0 (53.0–64.5)	0.9	58 (52.0–65.0)	0.5	0.5
Male sex	36 (90.0%)	37 (92.5%)	0.7	18 (51.4%)	< .001	< .001
African descent	5 (12.5%)	1 (2.5%)	0.09	n.a.		
MSM	32 (80.0%)	30 (75.0%)	0.8	n.a.		
CMV	38 (95.0%)	31 (77.5%)	0.02	8 (22.9%)	< .001	< .001
anti-CMV IgG	50.9 (23.5–108.6)	23.9 (13.8–87.8)	0.03	11.3 (10.2–16.8)	0.002	0.09
High avidity anti-CMV IgG	30.7 (13.0–57.0)	13.3 (8.2–39.7)	0.048	10.7 (10.0–13.2)	0.046	0.4
HBV	21 (52.5%)	7 (17.5%)	0.001	n.a.		
Cleared	18 (45.0%)	7 (17.5%)	0.008	n.a.		
Chronic	3 (7.5%)	0 (0%)	0.08	0 (0%)	0.1	n.d.
HCV (chronic and acute)	6 (15%)	0 (0%)	0.01	0 (0%)	0.02	n.d.
CD4 counts, cells/μl	589 (470–800)	961 (759–1233)	< .001	n.a.		
CD8 counts, cells/μl	762 (636–1029)	488 (364–621)	< .001	n.a.		
CD4:CD8 ratio	0.80 (0.61–1.13)	1.95 (1.33–2.83)	< .001	n.a.		
CD4 nadir, cells/μl	180 (60–180)					
Years since HIV diagnosis	13.9 (9.1–18.7)					
Years since ART	12.2 (7.9–16.9)					
Years undetectable plasma viral load (<200 c/ml)^2^	8.0 (5.3–10.9)					

^1^ *p* value calculated using Student’s t-test, Mann-Whitney U test or Chi-Square test, or Wilcoxon’s rank-sum test where applicable.

^2^ The threshold was set at 200 c/ml to exclude incidental viral blips from the period in which plasma viral load was detectable.

Abbreviations: HIV, human immunodeficiency virus; BBD, blood bank donors n.a., not available; n.d., not determined; IQR, interquartile range; MSM, men who have sex with men; CMV, Cytomegalovirus; HBV, hepatitis B virus; HCV, hepatitis C virus; ART, antiretroviral therapy.

### T cell activation and exhaustion in COBRA participants and blood bank donors

**Fig 1:**
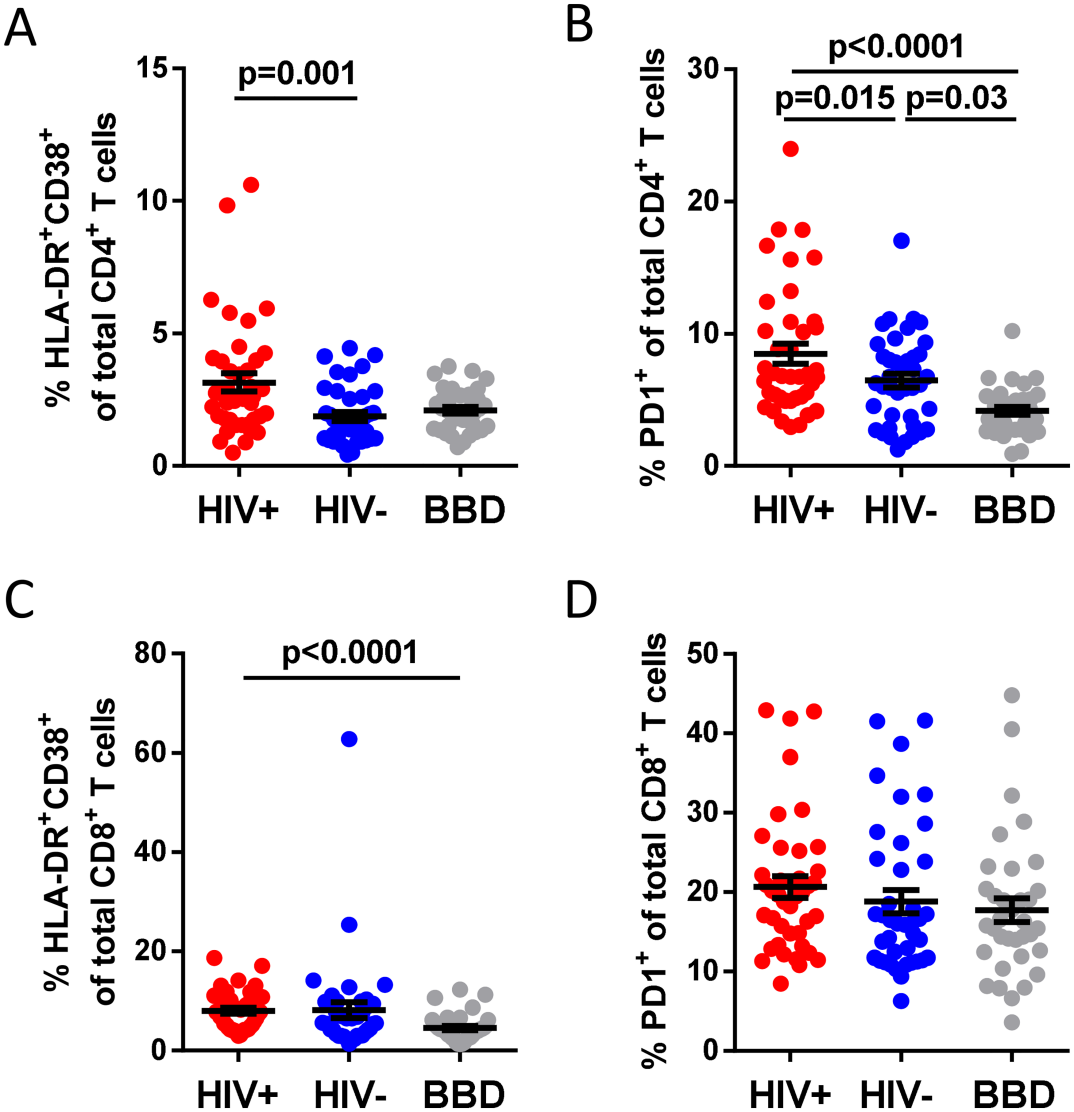
CD4 and CD8 T cell activation and exhaustion in HIV-positive and HIV-negative COBRA participants, and blood bank donors. (a) The percentage of activated (HLA-DR^+^CD38^+^) and (b) exhausted (PD-1^+^) CD4^+^ T cells within the CD4^+^ T-cell population. (c) The percentage of activated (HLA-DR^+^CD38^+^) and (d) exhausted (PD-1^+^) T cells within the CD8^+^ T-cell population. Significance was assessed with multivariable linear regression, corrected for age and gender. HIV+, HIV-positive; HIV-, HIV-negative; BBD, blood bank donors.

The percentages of activated (HLA-DR^+^CD38^+^) CD4 T cells and exhausted (PD1^+^) CD4 T cells were higher in ART-treated HIV-positive COBRA participants compared to both cohorts of HIV-negative participants (Table 2, Fig 1A and 1B). Interestingly, the percentage of PD1^+^ expressing CD4 T cells was higher in both the HIV-positive and HIV-negative COBRA participants when compared to the blood bank donors (Fig 1B and Table 2). The percentage of activated (HLA-DR^+^CD38^+^) CD8 T cells was higher in the HIV-positive COBRA participants but not in the HIV-negative COBRA participants when compared to the blood bank donors (Fig 1C and Table 2). No differences were observed in the percentage of exhausted (PD1^+^) CD8 T cells between the three groups (Fig 1D and Table 2).

**Table 2:** T cell activation, exhaustion and differentiation in HIV-positive and HIV-negative COBRA participants and blood bank donors.

Markers	HIV-positive median (IQR)	HIV-negative median (IQR)	HIV-pos vs HIV-neg *p* value^1^	BBD median (IQR)	BBD vs HIV-pos *p* value^1^	BBD vs HIV-neg *p* value^1^
**CD4**^**+**^ **T cells**						
% HLA-DR^+^CD38^+^ of CD4^+^ T cells^2^	2.58 (1.73–3.96)	1.56 (1.03–2.57)	0.001	2.09 (1.41–2.65)	0.06	0.06
% PD1^+^ of CD4^+^ T cells ^2^	6.77 (5.16–10.69)	6.2 (3.38–8.36)	0.015	4.2 (2.73–5.03)	2.0E-06	0.03
% CD57^+^ of CD4^+^ T cells ^2^	12.14 (6.32–18.43)	12.21 (7.01–19.31)	0.8	4.73 (2.91–8.47)	3.2E-04	0.001
% CD27^−^CD28^−^ of CD4^+^ T cells ^2^	4.76 (1.14–8.49)	6.03 (1.46–12.25)	0.8	0.19 (0.05–0.57)	1.0E-06	3.0E-06
% CD57^+^ of CD4^+^CD28^−^ T cells	58.88 (37.64–81.46)	79.59 (45.93–85.51)	0.07	32.23 (15.29–63.46)	0.015	2.9E-04
% CD45RA^+^CCR7^+^CD27^+^ of CD4^+^ T cells ^4^	11.4 (4.9–20.01)	18.54 (9.62–29.43)	0.017	23.37 (13.06–40.8)	9.4E-05	0.1
% CD45RA^−^CCR7^+^CD27^+^ of CD4^+^ T cells ^4^	16.6 (11.33–23.1)	15.99 (10.74–22.9)	0.5	19.4 (11.44–22.44)	0.5	0.4
% CD45RA^−^CCR7^−^CD27^+^ of CD4^+^ T cells ^2^	23.99 (21.17–33.27)	23.87 (18.75–27.1)	0.3	27.16 (19.81–33.86)	0.8	0.1
% CD45RA^−^CCR7^−^CD27^−^ of CD4^+^ T cells ^2^	11.85 (7.57–24.88)	10.55 (6.57–18.93)	0.4	6.97 (3.87–9.37)	.001	0.046
% CD45RA^+^CCR7^−^CD27^−^ of CD4^+^ T cells ^2^	0.93 (0.55–2.52)	2.05 (1.06–4.41)	0.1	0.74 (0.3–1.33)	0.2	0.039
**CD8**^**+**^ **T cells**						
% HLA-DR^+^CD38^+^ of CD8^+^ T cells ^2^	7.46 (4.54–10.8)	5.6 (3.29–9.65)	0.2	4.05 (2.66–5.68)	9.7E-05	0.051
% PD1^+^ of CD8^+^ T cells ^2^	19.58 (14.06–23.91)	16.08 (11.56–24.02)	0.2	15.79 (12.42–20.41)	0.4	0.9
% CD57^+^ of CD8^+^ T cells	50.6 (36.81–57.2)	45.08 (35.57–57.44)	0.8	30.4 (14.91–42.4)	.009	.031
% CD27^−^CD28^−^ of CD8^+^ T cells	38.1 (24.8–46.4)	36.4 (22.55–50.05)	0.7	12.2 (5.56–20.7)	4.5E-06	3.4E-04
% CD57^+^ of CD8^+^CD28^−^ T cells ^3^	75.75 (67.06–80.98)	79.31 (74.03–85.81)	0.045	61.4 (49.4–83.37)	0.6	0.037
% CD45RA^+^CCR7^+^CD27^+^ of CD8^+^ T cells ^4^	6.31 (3.67–10.12)	8.36 (4.19–15.23)	0.08	18.46 (9.85–30.48)	1.3E-05	0.025
% CD45RA^−^CCR7^+^CD27^+^ of CD8^+^ T cells ^2^	3.71 (2.81–5.69)	4.47 (1.87–7.61)	1.0	5.34 (3.08–8.79)	0.8	1.0
% CD45RA^−^CCR7^−^CD27^+^ of CD8^+^ T cells ^4^	26.8 (19.12–33.19)	20.72 (15.22–27.11)	0.06	34.49 (29.81–42.38)	0.2	0.003
% CD45RA^−^CCR7^−^CD27^−^ of CD8^+^ T cells ^2^	19.18 (14.37–29.89)	12.9 (9.13–19.46)	0.006	9.23 (5.79–15.22)	0.001	0.3
% CD45RA^+^CCR7^−^CD27^−^ of CD8^+^ T cells ^4^	19.17 (12.96–24.8)	19.18 (11.26–36.78)	0.3	8.88 (5.44–20.4)	0.1	0.047

^1^ Multivariable linear regression, corrected for age and gender. Uncorrected p values are given. Bonferroni adjustment for multiple testing required a p value of <0.0025 for significance.

^2^ LOG transformed to obtain normality.

^3^ acrsine transformed to obtain normality.

^4^ square root transformed to obtain normality

Abbreviations: HIV, human immunodeficiency virus; BBD, blood bank donors; IQR, interquartile range.

### T cell differentiation in COBRA participants and blood bank donors

In the CD4 compartment, the percentage of naïve cells (CD45RA^+^CD27^+^CCR7^+^) was lower in the HIV-positive COBRA participants compared to the HIV-negative COBRA participants, but no differences were observed in the percentage of central memory (CD45RA^−^CCR7^+^CD27^+^), transitional memory (CD45RA^−^CCR7^−^CD27^+^), effector memory (CD45RA^−^CCR7^−^CD27^−^) and TEMRA cells (CD45RA^+^CCR7^−^CD27^−^) (Table 2). When compared to the blood bank donors, we observed that HIV-positive and HIV-negative COBRA participants had a higher percentage of effector memory cells (CD45RA^−^CCR7^−^CD27^−^), whereas an additional increase in the percentage of TEMRA cells (CD45RA^+^CCR7^−^CD27^−^) was observed in the HIV-negative COBRA participants (Table 2).

Within the CD8 compartment, the percentage of effector memory cells (CD45RA^−^CCR7^−^CD27^−^) was higher in HIV-positive COBRA participants compared to the HIV-negative COBRA participants, whereas no differences were observed in the other populations (Table 2). When compared to the blood bank donors, HIV-positive and HIV-negative COBRA participants had a lower percentage of naïve CD8 cells (CD45RA^+^CD27^+^CCR7^+^). The percentage of effector memory cells (CD45RA^−^CCR7^−^CD27^−^) was higher in HIV-positive, while the percentage of transitional memory (CD45RA^−^CCR7^−^CD27^+^) was lower and TEMRA cells (CD45RA^+^CCR7^−^CD27^−^) higher in HIV-negative COBRA participants as compared to the blood bank donors (Table 2).

### Percentage of terminally differentiated T cells is increased in COBRA participants compared to blood bank donors

**Fig 2:**
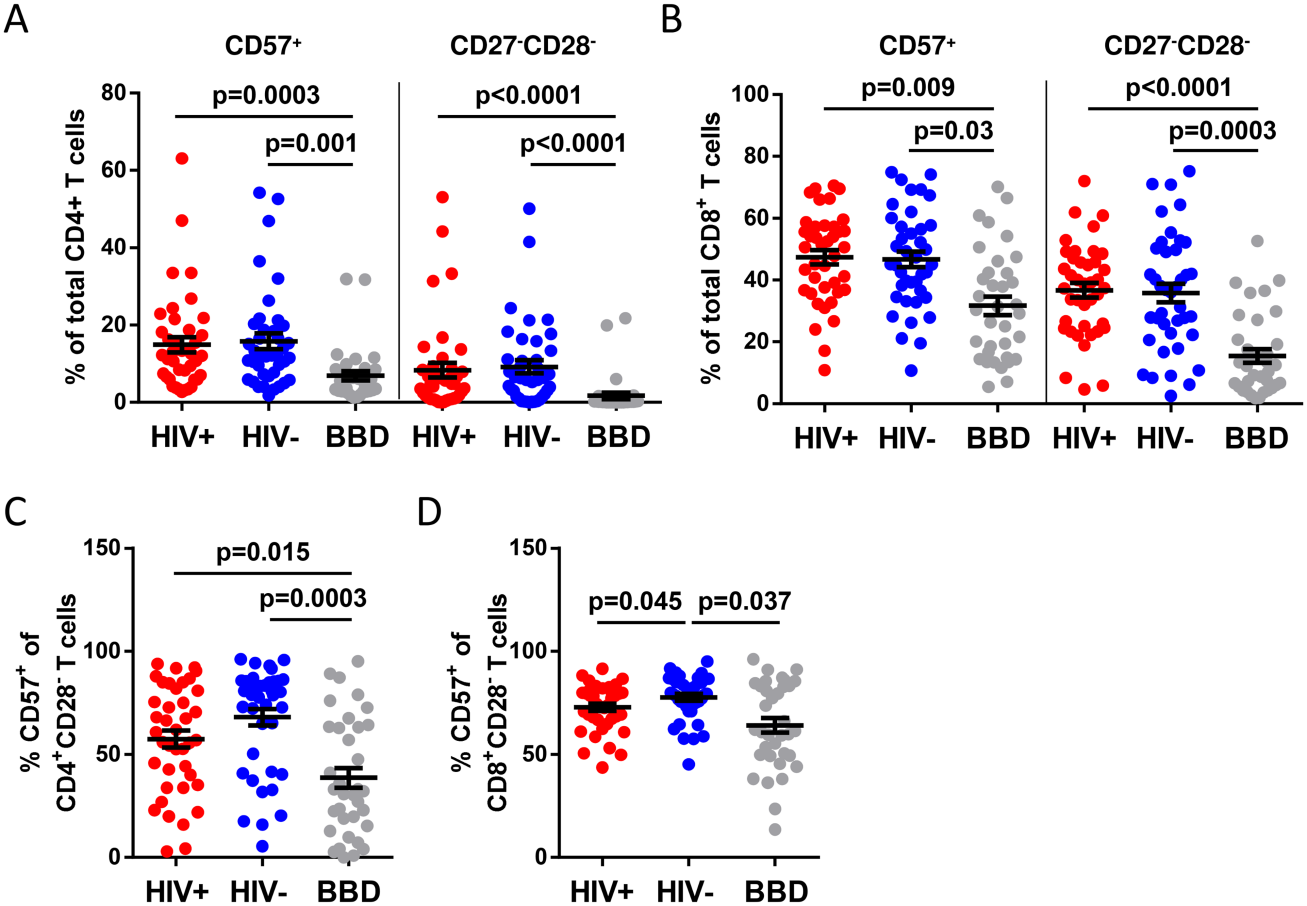
Terminally differentiated CD4 and CD8 T cells in HIV-positive and HIV-negative COBRA participants, and blood bank donors. (a) The percentage of CD57^+^ and CD27^−^CD28^−^ T cells within the CD4^+^ T cell population. (b) The percentage of CD57^+^ and CD27^−^CD28^−^ T cells within the CD8^+^ T cell population. (c) The percentage of CD57^+^ cells in the CD4^+^CD28^−^ and (d) CD8^+^CD28^−^cell population. Significance was assessed with multivariable linear regression, corrected for age and gender. HIV+, HIV-positive; HIV-, HIV-negative; BBD, blood bank donors.

No significant differences in the percentages of terminally differentiated T cells between the HIV-positive and HIV-negative COBRA participants were observed (Fig 2A and 2B and Table 2). Strikingly, a strong increase in the percentages of both terminally differentiated CD4 and CD8 T cells (CD57^+^ and CD27^−^/CD28^−^) was observed both when HIV-positive or HIV-negative COBRA participants were compared to blood bank donors (Fig 2A and 2B and Table 2). In addition, we analysed the percentage of CD57 expressing cells within the CD28^−^CD8^+^ T-cells or CD28^−^CD4^+^ T cells as these cells have been shown to accumulate upon chronic antigenic stimulation by viral pathogens [25]. The percentage of CD57 expressing cells within the CD28^−^CD4^+^ T cell population was not different between HIV-positive and HIV-negative COBRA participants (Fig 2C and Table 2). However when compared to the blood bank donors, the percentage of CD57 expressing cells within the CD28^−^CD4^+^ T cells was higher in both the HIV-positive and HIV-negative COBRA participants (Fig 2C and Table 2). The percentage of CD57 expressing cells within the CD28^−^CD8^+^ T cells was lower in HIV-positive COBRA participants as compared to HIV-negative COBRA participants (Fig 2D and Table 2). Blood bank donors have lower levels of CD57 expressing cells within the CD28^−^CD8^+^ T cells when compared to HIV-negative COBRA participants, but not when compared to HIV-positive COBRA participants (Table 2 and Fig 2D).

### HIV-1 and CMV infection are both associated with T cell activation, exhaustion and terminal differentiation

Multivariable linear regression analysis was performed to determine whether HIV-1 infection and CMV infection (CMV serostatus) were independently associated with T cell activation, exhaustion, and terminal differentiation of T cells. after adjusting for age and gender. Within the CD4 compartment, both T cell activation (HLA-DR^+^CD38^+^) and T cell exhaustion (PD1^+^) were higher in HIV-positive participants compared to HIV-negative COBRA participants, whereas T cell exhaustion was lower in blood bank donors as compared to HIV-negative COBRA participants (Table 3). Within the CD8 compartment, HIV infection was associated with higher T cell exhaustion while CMV infection was independently associated with lower CD8 T cell exhaustion (PD1^+^) (Table 3).

**Table 3:** Multivariable analyses of the association between HIV-1 infection, CMV serostatus, as well as CMV total and high avidity IgG titers, and T cell activation, exhaustion and terminal differentiation in HIV-positive and HIV-negative COBRA participants and blood bank donors.

			Complete population adjusted for CMV serostatus^1^		CMV positive participants adjusted for CMV Total IgG^2^		CMV positive participants adjusted for CMV High Avidity IgG^2^	
Activation and Exhaustion			B (95% CI)^5^	p value	B (95% CI) ^5^	p value	B (95% CI) ^5^	p value
% HLA-DR^+^CD38^+^ of CD4^+^ T cells^3^	Group	HIV-neg (ref)						
		HIV-pos	0.21 (0.10–0.32)	0.0003	0.18 (0.06–0.31)	0.005	0.19 (0.06–0.31)	0.003
		BBD	0.10 (-0.038–0.24)	0.2	0.07 (-0.15–0.30)	0.5	0.07 (-0.15–0.29)	0.5
	CMV		0.01 (-0.12–0.13)	0.9	0.001 (0.0003–0.002)	0.006	0.002 (0.0007–0.003)	0.003
% PD1^+^ of CD4^+^ T cells^3^	Group	HIV-neg (ref)						
		HIV-pos	0.13 (0.02–0.24)	0.02	0.15 (0.04–0.26)	0.01	0.16 (0.04–0.27)	0.007
		BBD	-0.14 (-0.27–0.01)	0.04	-0.06 (-0.26–0.14)	0.6	-0.06 (-0.26–0.14)	0.5
	CMV		-0.002 (-0.12–0.12)	1.0	0.0003 (-0.0003–0.001)	0.3	0.0002 (-0.0009–0.001)	0.7
% HLA-DR+CD38+ of CD8+ T cells^3^	Group	HIV-neg (ref)						
		HIV-pos	0.07 (-0.05–0.19)	0.3	0.03 (-0.11–0.17)	0.7	0.03 (-0.10–0.17)	0.6
		BBD	-0.11 (-0.26–0.03)	0.1	-0.23 (-0.47–0.01)	0.06	-0.23 (-0.48–0.009)	0.06
	CMV		0.07 (-0.07–0.21)	0.3	0.00005 (-0.0003–0.001)	0.2	0.0008 (-0.0005–0.002)	0.2
% PD1+ of CD8+ T cells^3^	Group	HIV-neg (ref)						
		HIV-pos	0.08 (-0.008–0.17)	0.07	0.11 (0.03–0.19)	0.008	0.11 (0.03–0.19)	0.007
		BBD	-0.07 (-0.18–0.04)	0.2	0.01 (-0.13–0.15)	0.9	0.01 (-0.13–0.15)	0.9
	CMV		-0.14 (-0.24–0.04)	0.006	-0.0006 (-0.0005–0.0004)	0.8	-0.0002 (-0.001–0.0006)	0.7
**Terminal differentiation**								
% CD57^+^ of CD4^+^ T cells^3^	Group	HIV-neg (ref)						
		HIV-pos	-0.09 (-0.23–0.05)	0.2	-0.14 (-0.30–0.02)	0.08	-0.14 (-0.29–0.02)	0.09
		BBD	-0.13 (-0.30–0.04)	0.1	-0.16 (-0.44–0.13)	0.3	-0.16 (-0.45–0.12)	0.3
	CMV		0.36 (0.20–0.51)	1.3E-05	0.001 (0.00003–0.002)	0.04	0.001 (-0.0001–0.003)	0.07
% CD27-CD28- of CD4+ T cells^3^	Group	HIV-neg (ref)						
		HIV-pos	-0.28 (-0.54–0.03)	0.03	-0.33 (-0.62–0.05)	0.02	-0.32 (-0.61–0.03)	0.03
		BBD	-0.35 (-0.66–0.04)	0.03	-0.49 (-1.00–0.03)	0.06	-0.49 (-1.01–0.02)	0.06
	CMV		1.32 (1.03–1.61)	5.2E-15	0.002 (0.00005–0.003)	0.04	0.003 (-0.0002–0.006)	0.06
% CD57+ of CD8+ T cells	Group	HIV-neg (ref)						
		HIV-pos	-1.89 (-8.07–4.28)	0.5	-3.77 (-10.52–2.99)	0.3	-3.63 (-10.25–2.99)	0.3
		BBD	-1.13 (-8.82–6.56)	0.7	-5.91 (-17.90–6.08)	0.3	-6.03 (-17.87–5.81)	0.3
	CMV		16.02 (8.97–23.07)	1.6E-05	0.05 (0.016–0.09)	0.006	0.10 (0.04–0.17)	0.003
% CD27-CD28- of CD8+ T cells	Group	HIV-neg (ref)						
		HIV-pos	-2.64 (-8.65–3.37)	0.4	-6.07 (-12.98–0.85)	0.09	-5.72 (-12.59–1.15)	0.1
		BBD	-5.22 (-12.70–2.27)	0.2	-15.21 (-27.49–2.94)	0.02	-15.40 (-27.68–3.12)	0.02
	CMV		21.38 (14.51–28.23)	1.2E-08	0.05 (0.02–0.09)	0.007	0.09 (0.03–0.16)	0.008
% CD57+ of CD4+CD28- T cells	Group	HIV-neg (ref)						
		HIV-pos	-17.67 (-27.73–7.61)	0.001	-18.31 (-29.31–7.32)	0.001	-18.67 (-29.45–7.88)	0.001
		BBD	-7.59 (-20.13–4.95)	0.2	-7.09 (-26.61–12.42)	0.5	-7.05 (-26.335–12.24)	0.5
	CMV		39.74 (28.25–51.24)	4.5E-10	0.04 (-0.03–0.10)	0.2	0.10 (-0.01–0.20)	0.08
% CD57+ of CD8+CD28- T cells^4^	Group	HIV-neg (ref)						
		HIV-pos	-0.10 (-0.18–0.01)	0.03	-0.10 (-0.19–0.02)	0.02	-0.10 (-0.19–0.02)	0.02
		BBD	-0.05 (-0.16–0.06)	0.3	-0.07 (-0.23–0.08)	0.3	-0.07 (-0.23–0.08)	0.3
	CMV		0.13 (0.03–0.23)	0.01	0.0004 (-0.0001–0.0009)	0.1	0.001 (0.00007–0.002)	0.04

^1^ Multivariable linear regression adjusted for age and gender in HIV-positive (n = 40), HIV-negative (n = 40) and BBD (n = 35). Uncorrected p values are given. Bonferroni adjustment for multiple testing required a p value of <0.005 for significance.

^2^ Multivariable linear regression adjusted for age and gender in HIV-positive (n = 38), HIV-negative (n = 31) and BBD (n = 8) infected with CMV. Uncorrected p values are given. Bonferroni adjustment for multiple testing required a p value of <0.005 for significance.

^3^ LOG transformed to obtain normality.

^4^ acrsine transformed to obtain normality.

^5^Unstandardized coefficient (B) indicates the difference in the outcome (immunological markers) with respect to the variables (study group, CMV infection). For example, B is the increase in LOG transformed % of HLA-DR+CD38+ of CD4+ T cells in a HIV-positive individual as compared to a HIV-negative participant.

Abbreviations: HIV, human immunodeficiency virus; BBD, blood bank donors; B, unstandardized regression coefficient; CI, confidence interval; CMV, Cytomegalovirus; IgG, Immunoglobulin G

When we analysed markers of terminally differentiated T cells (CD57^+^ and CD27^−^CD28^−^) we observed that the percentage of CD27^−^CD28^−^ cells within CD4^+^ T cells was lower in both HIV-positive COBRA participants and blood bank donors when compared to HIV-negative COBRA participants (Table 3). Moreover, a strong association between a higher proportion of terminally differentiated (CD57^+^ and CD27^−^CD28^−^) CD4 and CD8 T cells and CMV infection was observed (Table 3). The percentages of CD57 expressing cells within the CD28^−^CD8^+^ T-cells and CD28^−^CD4^+^ T cells were lower in HIV-positive COBRA participants, while a positive association with CMV infection was observed.

### CMV IgG titers are associated with terminal differentiation of T cells and CD8 T cell exhaustion

Anti-CMV IgG antibodies were positive in 95% of the HIV-positive COBRA participants, 77.5% of the HIV-negative COBRA participants, and 22.9% of the blood bank donors. HIV-positive COBRA participants had higher levels of total and high avidity CMV-IgG antibody titers as compared to both the HIV-negative COBRA participants and the blood bank donors (Table 1), confirming previous observations [30, 32, 33]. Increased CMV IgG levels are suggestive of increased stimulation by CMV antigens, which may further contribute to T cell activation, exhaustion and terminal differentiation. Total CMV IgG was associated with higher CD4 T cell activation (HLA-DR^+^CD38^+^), and terminally differentiated (CD57^+^ and CD27^−^CD28^−^) CD4 and CD8 T cells in CMV-positive individuals irrespective of HIV-1 infection (Table 3). In this model, CD4 T cell activation (HLA-DR^+^CD38^+^), CD4 T cell exhaustion (PD1^+^) and CD8 T cell exhaustion (PD1^+^) were still higher in HIV-positive participants, whereas the percentage of CD4^+^CD27^−^CD28^−^ T cells, the percentage of CD57 expressing cells within the CD28^−^CD8^+^ T-cells and CD28^−^CD4^+^ T cells were lower in HIV-positive COBRA participants when compared to HIV-negative COBRA participants (Table 3). Similarly, CMV high avidity IgG levels were associated with higher CD4 T cell activation, terminally differentiated (CD57^+^ and CD27^−^CD28^−^) CD8 T cells, the percentage of CD57^+^ cells within CD8^+^CD28^−^ cells, but not terminally differentiated (CD57^+^ and CD27^−^CD28^−^) CD4 T cells (Table 3).

## Discussion

Despite effective ART, HIV-positive individuals are reported to have higher rates of age-associated non-communicable diseases and a shorter average life expectancy compared to uninfected persons of the same age [3, 5, 7]. The increased morbidity and mortality in the HIV-1 infected population may be the result of several contributing factors such as ART toxicity, chronic immune activation and immune dysfunction as well as a higher prevalence of traditional risk factors for these AANCC [8, 9]. Here we analysed markers of T cell activation, exhaustion, and differentiation in a cohort of HIV-positive adults who were on apparently effective ART and compared these to data from adults who were comparable for age, lifestyle and demographic factors (COBRA) and a cohort age matched blood bank donors. HIV-positive COBRA participants showed incomplete immune recovery as reflected by lower CD4 T cell counts and higher CD8 T cell counts than observed in either well-matched or just age-matched uninfected controls. Furthermore, a higher percentage of activated and exhausted CD4 cells, a lower percentage of CD4 naïve cells and higher percentage of CD8 effector memory cells were observed when compared to HIV-negative COBRA participants which is broadly consistent with previous studies [30, 34–40]. In contrast, measures of terminally differentiated T cells (CD57^+^ and CD27^−^ CD28^−^), did not differ between the treated HIV-positive and HIV-negative COBRA participants, confirming previous observations [17, 30], but contradicting other studies [8, 13, 25, 27]. Collectively, these data provide strong support for the concept that certain HIV-mediated immunologic perturbations (e.g., low CD4/CD8 ratio, shift towards more differentiated memory cell types, high PD-1 expression) do indeed persist indefinitely during ART, while other markers more typically associated with terminal differentiation and senescence may normalize, at least compared to levels observed in well-matched persons who lack HIV but have other risk factors, particularly CMV infection.

Strikingly, the percentages of activated CD8 T cells, exhausted CD4 T cells and terminally differentiated CD4 and CD8 T cells were higher in both HIV-positive and HIV-negative COBRA participants when compared to the general populations (e.g., the blood bank donors). HIV-positive and negative COBRA participants are more often infected with CMV when compared to the blood bank donors which may be due to the high number of men-who-have-sex-with-men in the COBRA participants. Multivariable analysis showed that HIV-1 infection is independently associated with CD4 T cell activation and T cell exhaustion but not with terminally differentiated T cells (CD57^+^ and CD27^−^CD28^−^). CMV infection was strongly associated with increased proportion of terminally differentiated T cells and may at least partially explain the higher levels of terminally differentiated T cells in the HIV-positive and HIV-negative COBRA participants. This is in line with previous publications in which CMV infection was strongly associated with increased terminal differentiation of T cells and CD8 T cell exhaustion [13, 15, 17, 28, 29]. However, no association of CMV infection with T cell activation was observed which is in contrast with previous studies [41–43]. HIV-positive individuals in our study are considered to be successfully treated, which is reflected in the high CD4 counts and relatively high CD4:CD8 ratios. Furthermore, they have a relative low % of activated CD8 cells (median 7,5% CD8^+^HLA-DR^+^CD38^+^) as compared to the other studies (15–20% CD8^+^HLA-DR^+^CD38^+^) [41–43], and this might at least in part explain the differences between the studies. We did however observe that higher CMV IgG titers were associated with higher CD4 T cell activation in the CMV-positive participants.

HIV-positive COBRA participants had increased levels of CMV specific IgG when compared to HIV-negative COBRA participants and blood bank donors, confirming previous observations [30, 32, 33]. CMV specific IgG levels are considered a surrogate marker of CMV reactivation and concurrent immune response to control infection, and high levels are associated with ageing and increased morbidity and mortality in both the general population and HIV-positive individuals [30–33, 44–46]. Total CMV IgG was independently associated with the presence of a higher proportion of terminally differentiated T cells and CD4 T cell activation, indicating that reactivation of CMV infection contributes substantially to terminal differentiation of T cells and CD4 activation in treated HIV-positive individuals. This could suggest that the high CMV prevalence combined with increased CMV reactivation may contribute to the increased comorbidity burden that has been reported in treated HIV-positive individuals [7]. Ageing and CMV infection are associated with terminal differentiation and proliferation of CD8^+^CD28^−^CD57^+^ effector memory cells, however HIV-1 has been shown to inhibit the terminal differentiation of these cells which is reflected in a decreased percentage of CD28^−^CD8^+^ T-cells expressing CD57 [17, 18]. In line with these findings we observed that HIV-1 infection was independently associated with a decrease in the percentage of CD57 expressing cells within the CD28^−^CD8^+^ T cells, whereas CMV infection was independently associated with an increased percentage of these cells.

Cigarette smoking is known to have an impact on different immune functions, with both proinflammatory and immune suppressive effects having been described [47, 48]. For example, increased levels of markers of inflammation (CRP, IL-6, D-dimer, sCD14), T cells activation (% HLA-DR^+^CD38^+^ of CD4 and CD8 cells), and decreased T cell function (% PD1^+^ CD4 and CD8 T cells) have been associated with smoking in HIV-infected individuals as well as in the general population [49–52]. In the present study, data about cigarette smoking was only available for the COBRA participants (HIV-positive and HIV-negative), and in this group no association between smoking and T cell activation, T cell exhaustion, and terminal differentiation of CD4 and CD8 cells was observed (data not shown). However, we cannot exclude that the limited number of individuals analysed and the lack of data about cigarette smoking in the BBD group might explain why previously reported associations between cigarette smoking and T cell activation and function could not be confirmed [49, 51].

Due to its observational nature, our study does suffer from several limitations. Firstly, although we attempted to identify independent effects of HIV and CMV, CMV infection was highly prevalent in both groups of COBRA participants, reflecting the high numbers of MSM in each group (for example, only 2 of the 40 HIV-positive participants were CMV-negative). Thus our ability to differentiate the independent effects of the two viruses is limited. For this reason, analyses of CMV IgG may provide greater discriminative ability. Secondly, we cannot rule out the possibility that the effects seen were a consequence of other unmeasured differences between the groups rather than HIV and CMV infection per se. Finally, the small numbers in some of our groups, and the low prevalence of coinfection with HBV and HCV meant that we were unable to assess the effects of coinfection with these viruses.

In conclusion, HIV-positive individuals on ART with long-term suppressed viraemia exhibited incomplete immune recovery and increased immune activation/exhaustion compared to HIV-negative controls matched for age, lifestyle and demographic factors. However, no evidence for increased immune senescence as determined by the level of terminally differentiated T cells, was observed when HIV-positive individuals on suppressive ART were compared to appropriately selected controls matched for age, lifestyle and demographic factors. If only blood donors or perhaps someone poorly matched convenience sample had been used as controls one would have falsely concluded that HIV was independently associated with increased proportion of terminally differentiated T cells. This illustrates the importance of recruiting and utilising appropriate control populations for such studies.

## Supporting information

S1 Fig Gating strategy flow cytometry. **A**. Gating of CD4+ and CD8+ T cells. **B**. T cell differentiation was defined as the proportion of naïve (N; CD45RA^+^CD27^+^CCR7^+^), central memory (CM; CD45RA^−^CCR7^+^CD27^+^), transitional memory (TM; CD45RA^−^CCR7^−^CD27^+^), effector memory (EM; CD45RA^−^CCR7^−^CD27^−^), and terminally differentiated effector memory (TEMRA; CD45RA^+^CCR7^−^CD27^−^) within the total CD4 or CD8 T cell population. **C**. T cell activation was defined as the proportion of cells that were positive for both CD38 and HLA-DR within the total CD4 or CD8 T cell population. T cell exhaustion was defined as the proportion of PD1 positive cells within the total CD4 or CD8 T cell population. Terminally differentiated T cells were defined as proportion of CD57 positive cells within the total CD4 or CD8 T cells population, the proportion of cells negative for both CD27 and CD28 within the total CD4 or CD8 T-cells population, or the proportion of CD57 positive within the CD28^−^CD4^+^ or CD28^−^CD8^+^ T cell population.
(PDF)

## References

[1] Wada N, Jacobson LP, Cohen M, French A, Phair J, Munoz A. Cause-specific life expectancies after 35 years of age for human immunodeficiency syndrome-infected and human immunodeficiency syndrome-negative individuals followed simultaneously in long-term cohort studies, 1984–2008. Am J Epidemiol. 2013;177(2):116–25. doi: 10.1093/aje/kws321 ; 23287403 10.1093/aje/kws321 PMC3590031

[2] Weber R, Ruppik M, Rickenbach M, Spoerri A, Furrer H, Battegay M, et al Decreasing mortality and changing patterns of causes of death in the Swiss HIV Cohort Study. HIV Med. 2013;14(4):195–207. doi: 10.1111/j.1468-1293.2012.01051.x . 22998068 10.1111/j.1468-1293.2012.01051.x

[3] Guaraldi G, Orlando G, Zona S, Menozzi M, Carli F, Garlassi E, et al Premature age-related comorbidities among HIV-infected persons compared with the general population. Clin Infect Dis. 2011;53(11):1120–6. doi: 10.1093/cid/cir627 . 21998278 10.1093/cid/cir627

[4] Effros RB, Fletcher CV, Gebo K, Halter JB, Hazzard WR, Horne FM, et al Aging and infectious diseases: workshop on HIV infection and aging: what is known and future research directions. Clin Infect Dis. 2008;47(4):542–53. ; 18627268 10.1086/590150 PMC3130308

[5] Hasse B, Ledergerber B, Furrer H, Battegay M, Hirschel B, Cavassini M, et al Morbidity and aging in HIV-infected persons: the Swiss HIV cohort study. Clin Infect Dis. 2011;53(11):1130–9. doi: 10.1093/cid/cir626 . 21998280 10.1093/cid/cir626

[6] Pathai S, Bajillan H, Landay AL, High KP. Is HIV a model of accelerated or accentuated aging? J Gerontol A Biol Sci Med Sci. 2014;69(7):833–42. doi: 10.1093/gerona/glt168 ; 24158766 10.1093/gerona/glt168 PMC4067117

[7] Schouten J, Wit FW, Stolte IG, Kootstra NA, van der Valk M, Geerlings SE, et al Cross-sectional comparison of the prevalence of age-associated comorbidities and their risk factors between HIV-infected and uninfected individuals: the AGEhIV cohort study. Clin Infect Dis. 2014;59(12):1787–97. doi: 10.1093/cid/ciu701 . 25182245 10.1093/cid/ciu701

[8] Deeks SG. HIV infection, inflammation, immunosenescence, and aging. Annu Rev Med. 2011;62:141–55. doi: 10.1146/annurev-med-042909-093756 ; 21090961 10.1146/annurev-med-042909-093756 PMC3759035

[9] Deeks SG, Tracy R, Douek DC. Systemic effects of inflammation on health during chronic HIV infection. Immunity. 2013;39(4):633–45. doi: 10.1016/j.immuni.2013.10.001 ; 24138880 10.1016/j.immuni.2013.10.001 PMC4012895

[10] Tenorio AR, Zheng Y, Bosch RJ, Krishnan S, Rodriguez B, Hunt PW, et al Soluble markers of inflammation and coagulation but not T-cell activation predict non-AIDS-defining morbid events during suppressive antiretroviral treatment. J Infect Dis. 2014;210(8):1248–59. doi: 10.1093/infdis/jiu254 ; 24795473 10.1093/infdis/jiu254 PMC4192039

[11] McComsey GA, Kitch D, Sax PE, Tierney C, Jahed NC, Melbourne K, et al Associations of inflammatory markers with AIDS and non-AIDS clinical events after initiation of antiretroviral therapy: AIDS clinical trials group A5224s, a substudy of ACTG A5202. J Acquir Immune Defic Syndr. 2014;65(2):167–74. doi: 10.1097/01.qai.0000437171.00504.41 ; 24121755 10.1097/01.qai.0000437171.00504.41 PMC3943548

[12] Karim R, Mack WJ, Kono N, Tien PC, Anastos K, Lazar J, et al T-cell activation, both pre- and post-HAART levels, correlates with carotid artery stiffness over 6.5 years among HIV-infected women in the WIHS. J Acquir Immune Defic Syndr. 2014;67(3):349–56. doi: 10.1097/QAI.0000000000000311 ; 25314253 10.1097/QAI.0000000000000311 PMC4197806

[13] Appay V, Fastenackels S, Katlama C, Ait-Mohand H, Schneider L, Guihot A, et al Old age and anti-cytomegalovirus immunity are associated with altered T-cell reconstitution in HIV-1-infected patients. AIDS. 2011;25(15):1813–22. doi: 10.1097/QAD.0b013e32834640e6 . 21412126 10.1097/QAD.0b013e32834640e6

[14] Appay V, Dunbar PR, Callan M, Klenerman P, Gillespie GM, Papagno L, et al Memory CD8+ T cells vary in differentiation phenotype in different persistent virus infections. Nat Med. 2002;8(4):379–85. doi: 10.1038/nm0402-379 . 11927944 10.1038/nm0402-379

[15] Kalayjian RC, Landay A, Pollard RB, Taub DD, Gross BH, Francis IR, et al Age-related immune dysfunction in health and in human immunodeficiency virus (HIV) disease: association of age and HIV infection with naive CD8+ cell depletion, reduced expression of CD28 on CD8+ cells, and reduced thymic volumes. J Infect Dis. 2003;187(12):1924–33. doi: 10.1086/375372 . 12792869 10.1086/375372

[16] Le Priol Y, Puthier D, Lecureuil C, Combadiere C, Debre P, Nguyen C, et al High cytotoxic and specific migratory potencies of senescent CD8+ CD57+ cells in HIV-infected and uninfected individuals. J Immunol. 2006;177(8):5145–54. . 17015699 10.4049/jimmunol.177.8.5145

[17] Lee SA, Sinclair E, Hatano H, Hsue PY, Epling L, Hecht FM, et al Impact of HIV on CD8+ T cell CD57 expression is distinct from that of CMV and aging. PLoS One. 2014;9(2):e89444 doi: 10.1371/journal.pone.0089444 ; 24586783 10.1371/journal.pone.0089444 PMC3937334

[18] Lee SA, Sinclair E, Jain V, Huang Y, Epling L, Van Natta M, et al Low proportions of CD28- CD8+ T cells expressing CD57 can be reversed by early ART initiation and predict mortality in treated HIV infection. J Infect Dis. 2014;210(3):374–82. doi: 10.1093/infdis/jiu109 ; 24585893 10.1093/infdis/jiu109 PMC4110459

[19] Franceschi C, Monti D, Barbieri D, Salvioli S, Grassilli E, Capri M, et al Successful immunosenescence and the remodelling of immune responses with ageing. Nephrol Dial Transplant. 1996;11 Suppl 9:18–25. . 9050030 10.1093/ndt/11.supp9.18

[20] Franceschi C, Bonafe M, Valensin S. Human immunosenescence: the prevailing of innate immunity, the failing of clonotypic immunity, and the filling of immunological space. Vaccine. 2000;18(16):1717–20. . 10689155 10.1016/s0264-410x(99)00513-7

[21] Deeks SG, Phillips AN. HIV infection, antiretroviral treatment, ageing, and non-AIDS related morbidity. BMJ. 2009;338:a3172 doi: 10.1136/bmj.a3172 . 19171560 10.1136/bmj.a3172

[22] Deeks SG. Immune dysfunction, inflammation, and accelerated aging in patients on antiretroviral therapy. Top HIV Med. 2009;17(4):118–23. . 19890183

[23] Skiest DJ, Rubinstien E, Carley N, Gioiella L, Lyons R. The importance of comorbidity in HIV-infected patients over 55: a retrospective case-control study. Am J Med. 1996;101(6):605–11. doi: 10.1016/S0002-9343(96)00329-4 . 9003107 10.1016/S0002-9343(96)00329-4

[24] Naeger DM, Martin JN, Sinclair E, Hunt PW, Bangsberg DR, Hecht F, et al Cytomegalovirus-specific T cells persist at very high levels during long-term antiretroviral treatment of HIV disease. PLoS One. 2010;5(1):e8886 doi: 10.1371/journal.pone.0008886 ; 20126452 10.1371/journal.pone.0008886 PMC2813282

[25] Dock JN, Effros RB. Role of CD8 T Cell Replicative Senescence in Human Aging and in HIV-mediated Immunosenescence. Aging Dis. 2011;2(5):382–97. ; 22308228 PMC3269814

[26] Grady BP, Nanlohy NM, van Baarle D. HCV monoinfection and HIV/HCV coinfection enhance T-cell immune senescence in injecting drug users early during infection. Immun Ageing. 2016;13:10 doi: 10.1186/s12979-016-0065-0 ; 27034702 10.1186/s12979-016-0065-0 PMC4815107

[27] Brenchley JM, Karandikar NJ, Betts MR, Ambrozak DR, Hill BJ, Crotty LE, et al Expression of CD57 defines replicative senescence and antigen-induced apoptotic death of CD8+ T cells. Blood. 2003;101(7):2711–20. doi: 10.1182/blood-2002-07-2103 . 12433688 10.1182/blood-2002-07-2103

[28] Freeman ML, Mudd JC, Shive CL, Younes SA, Panigrahi S, Sieg SF, et al CD8 T-Cell Expansion and Inflammation Linked to CMV Coinfection in ART-treated HIV Infection. Clin Infect Dis. 2016;62(3):392–6. doi: 10.1093/cid/civ840 ; 26400999 10.1093/cid/civ840 PMC4706630

[29] Tassiopoulos K, Landay A, Collier AC, Connick E, Deeks SG, Hunt P, et al CD28-negative CD4+ and CD8+ T cells in antiretroviral therapy-naive HIV-infected adults enrolled in adult clinical trials group studies. J Infect Dis. 2012;205(11):1730–8. doi: 10.1093/infdis/jis260 ; 22448010 10.1093/infdis/jis260 PMC3415854

[30] Cobos Jimenez V, Wit FW, Joerink M, Maurer I, Harskamp AM, Schouten J, et al T-Cell Activation Independently Associates With Immune Senescence in HIV-Infected Recipients of Long-term Antiretroviral Treatment. J Infect Dis. 2016 doi: 10.1093/infdis/jiw146 . 27073222 10.1093/infdis/jiw146 PMC8445638

[31] Klenerman P, Oxenius A. T cell responses to cytomegalovirus. Nat Rev Immunol. 2016 doi: 10.1038/nri.2016.38 . 27108521 10.1038/nri.2016.38

[32] Parrinello CM, Sinclair E, Landay AL, Lurain N, Sharrett AR, Gange SJ, et al Cytomegalovirus immunoglobulin G antibody is associated with subclinical carotid artery disease among HIV-infected women. J Infect Dis. 2012;205(12):1788–96. doi: 10.1093/infdis/jis276 ; 22492856 10.1093/infdis/jis276 PMC3415890

[33] Brunt SJ, Lee S, D'Orsogna L, Bundell C, Burrows S, Price P. The use of humoral responses as a marker of CMV burden in HIV patients on ART requires consideration of T-cell recovery and persistent B-cell activation. Dis Markers. 2014;2014:947432 doi: 10.1155/2014/947432 ; 25506120 10.1155/2014/947432 PMC4259131

[34] Cockerham LR, Siliciano JD, Sinclair E, O'Doherty U, Palmer S, Yukl SA, et al CD4+ and CD8+ T cell activation are associated with HIV DNA in resting CD4+ T cells. PLoS One. 2014;9(10):e110731 doi: 10.1371/journal.pone.0110731 ; 25340755 10.1371/journal.pone.0110731 PMC4207702

[35] Chevalier MF, Petitjean G, Dunyach-Remy C, Didier C, Girard PM, Manea ME, et al The Th17/Treg ratio, IL-1RA and sCD14 levels in primary HIV infection predict the T-cell activation set point in the absence of systemic microbial translocation. PLoS Pathog. 2013;9(6):e1003453 doi: 10.1371/journal.ppat.1003453 ; 23818854 10.1371/journal.ppat.1003453 PMC3688532

[36] Breton G, Chomont N, Takata H, Fromentin R, Ahlers J, Filali-Mouhim A, et al Programmed death-1 is a marker for abnormal distribution of naive/memory T cell subsets in HIV-1 infection. J Immunol. 2013;191(5):2194–204. doi: 10.4049/jimmunol.1200646 ; 23918986 10.4049/jimmunol.1200646 PMC3815464

[37] Said EA, Dupuy FP, Trautmann L, Zhang Y, Shi Y, El-Far M, et al Programmed death-1-induced interleukin-10 production by monocytes impairs CD4+ T cell activation during HIV infection. Nat Med. 2010;16(4):452–9. doi: 10.1038/nm.2106 ; 20208540 10.1038/nm.2106 PMC4229134

[38] Lim A, Tan D, Price P, Kamarulzaman A, Tan HY, James I, et al Proportions of circulating T cells with a regulatory cell phenotype increase with HIV-associated immune activation and remain high on antiretroviral therapy. AIDS. 2007;21(12):1525–34. doi: 10.1097/QAD.0b013e32825eab8b . 17630546 10.1097/QAD.0b013e32825eab8b

[39] Piconi S, Trabattoni D, Gori A, Parisotto S, Magni C, Meraviglia P, et al Immune activation, apoptosis, and Treg activity are associated with persistently reduced CD4+ T-cell counts during antiretroviral therapy. AIDS. 2010;24(13):1991–2000. doi: 10.1097/QAD.0b013e32833c93ce . 20651586 10.1097/QAD.0b013e32833c93ce

[40] Weiss L, Piketty C, Assoumou L, Didier C, Caccavelli L, Donkova-Petrini V, et al Relationship between regulatory T cells and immune activation in human immunodeficiency virus-infected patients interrupting antiretroviral therapy. PLoS One. 2010;5(7):e11659 doi: 10.1371/journal.pone.0011659 ; 20657770 10.1371/journal.pone.0011659 PMC2908121

[41] Gianella S, Massanella M, Richman DD, Little SJ, Spina CA, Vargas MV, et al Cytomegalovirus replication in semen is associated with higher levels of proviral HIV DNA and CD4+ T cell activation during antiretroviral treatment. J Virol. 2014;88(14):7818–27. doi: 10.1128/JVI.00831-14 ; 24789781 10.1128/JVI.00831-14 PMC4097769

[42] Hunt PW, Martin JN, Sinclair E, Epling L, Teague J, Jacobson MA, et al Valganciclovir reduces T cell activation in HIV-infected individuals with incomplete CD4+ T cell recovery on antiretroviral therapy. J Infect Dis. 2011;203(10):1474–83. doi: 10.1093/infdis/jir060 ; 21502083 10.1093/infdis/jir060 PMC3080892

[43] Wittkop L, Bitard J, Lazaro E, Neau D, Bonnet F, Mercie P, et al Effect of cytomegalovirus-induced immune response, self antigen-induced immune response, and microbial translocation on chronic immune activation in successfully treated HIV type 1-infected patients: the ANRS CO3 Aquitaine Cohort. J Infect Dis. 2013;207(4):622–7. doi: 10.1093/infdis/jis732 . 23204178 10.1093/infdis/jis732

[44] Roberts ET, Haan MN, Dowd JB, Aiello AE. Cytomegalovirus antibody levels, inflammation, and mortality among elderly Latinos over 9 years of follow-up. Am J Epidemiol. 2010;172(4):363–71. doi: 10.1093/aje/kwq177 ; 20660122 10.1093/aje/kwq177 PMC2950794

[45] Gkrania-Klotsas E, Langenberg C, Sharp SJ, Luben R, Khaw KT, Wareham NJ. Higher immunoglobulin G antibody levels against cytomegalovirus are associated with incident ischemic heart disease in the population-based EPIC-Norfolk cohort. J Infect Dis. 2012;206(12):1897–903. doi: 10.1093/infdis/jis620 . 23045624 10.1093/infdis/jis620

[46] Lichtner M, Cicconi P, Vita S, Cozzi-Lepri A, Galli M, Lo Caputo S, et al Cytomegalovirus coinfection is associated with an increased risk of severe non-AIDS-defining events in a large cohort of HIV-infected patients. J Infect Dis. 2015;211(2):178–86. doi: 10.1093/infdis/jiu417 . 25081936 10.1093/infdis/jiu417

[47] Holt PG. Immune and inflammatory function in cigarette smokers. Thorax. 1987;42(4):241–9. ; 3303428 10.1136/thx.42.4.241 PMC460693

[48] Stampfli MR, Anderson GP. How cigarette smoke skews immune responses to promote infection, lung disease and cancer. Nat Rev Immunol. 2009;9(5):377–84. doi: 10.1038/nri2530 . 19330016 10.1038/nri2530

[49] de Heens GL, van der Velden U, Loos BG. Cigarette smoking enhances T cell activation and a Th2 immune response; an aspect of the pathophysiology in periodontal disease. Cytokine. 2009;47(3):157–61. doi: 10.1016/j.cyto.2009.05.006 . 19616447 10.1016/j.cyto.2009.05.006

[50] Kooij KW, Wit FW, Booiman T, van der Valk M, Schim van der Loeff MF, Kootstra NA, et al Cigarette Smoking and Inflammation, Monocyte Activation, and Coagulation in HIV-Infected Individuals Receiving Antiretroviral Therapy, Compared With Uninfected Individuals. J Infect Dis. 2016;214(12):1817–21. doi: 10.1093/infdis/jiw459 . 27683822 10.1093/infdis/jiw459

[51] Valiathan R, Miguez MJ, Patel B, Arheart KL, Asthana D. Tobacco smoking increases immune activation and impairs T-cell function in HIV infected patients on antiretrovirals: a cross-sectional pilot study. PLoS One. 2014;9(5):e97698 doi: 10.1371/journal.pone.0097698 ; 24842313 10.1371/journal.pone.0097698 PMC4026405

[52] Yanbaeva DG, Dentener MA, Creutzberg EC, Wesseling G, Wouters EF. Systemic effects of smoking. Chest. 2007;131(5):1557–66. doi: 10.1378/chest.06-2179 . 17494805 10.1378/chest.06-2179

