# Supplementary material for: Terminal differentiation of T cells is strongly associated with CMV infection and increased in HIV-positive individuals on ART and lifestyle matched controls
Source: PLoS One. 2017 Aug 14;12(8):e0183357. doi: 10.1371/journal.pone.0183357 (PMC5555623; doi:10.1371/journal.pone.0183357)
Supplement: S1 Fig — A. Gating of CD4+ and CD8+ T cells. B. T cell differentiation was defined as the proportion of naïve (N; CD45RA+CD27+CCR7+), central memory (CM; CD45RA−CCR7+CD27+), transitional memory (TM; CD45RA−CCR7−CD27+), effector memory (EM; CD45RA−CCR7−CD27−), and terminally differentiated effector memory (TEMRA; CD45RA+CCR7−CD27−) within the total CD4 or CD8 T cell population. C. T cell activation was defined as the proportion of cells that were positive for both CD38 and HLA-DR within the total CD4 or CD8 T cell population. T cell exhaustion was defined as the proportion of PD1 positive cells within the total CD4 or CD8 T cell population. Terminally differentiated T cells were defined as proportion of CD57 positive cells within the total CD4 or CD8 T cells population, the proportion of cells negative for both CD27 and CD28 within the total CD4 or CD8 T-cells population, or the proportion of CD57 positive within the CD28−CD4+ or CD28−CD8+ T cell population. (PDF) [file pone.0183357.s001.pdf]

# S1 Fig.

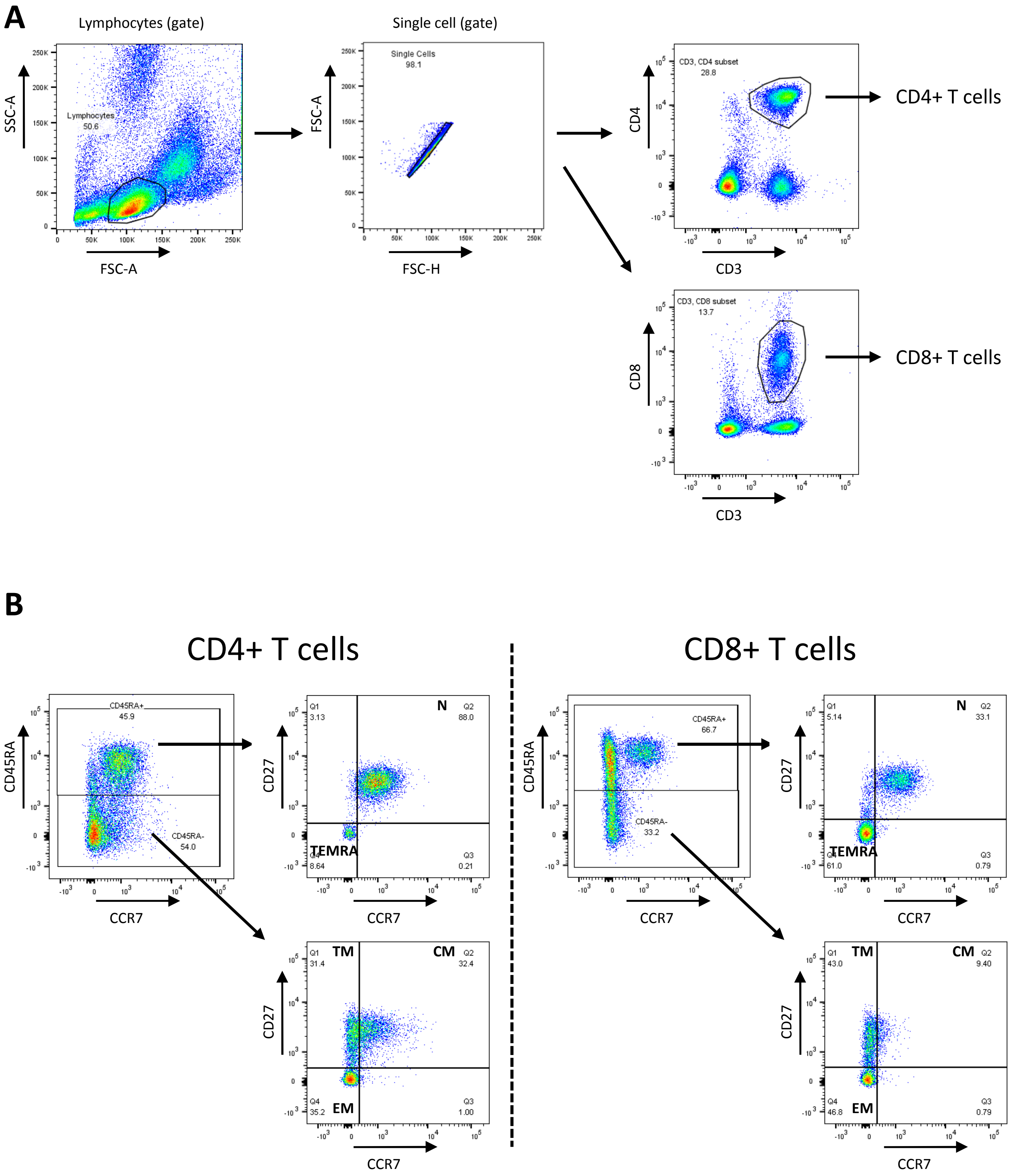

A
Lymphocytes (gate)
Single cell (gate)
Lymphocytes
50.6
SSC-A
FSC-A
Single Cells
98.1
FSC-H
CD3, CD4 subset
28.8
CD4
CD3
CD4+ T cells
CD3, CD8 subset
13.7
CD8
CD3
CD8+ T cells
B
CD4+ T cells
CD8+ T cells
CD45RA+
45.9
CD45RA-
54.0
CD45RA
CCR7
Q1
3.13
N
Q2
88.0
TEMRA
8.64
Q3
0.21
CD27
CCR7
Q1
TM
31.4
CM
Q2
32.4
Q4
EM
35.2
Q3
1.00
CD27
CCR7
CD45RA+
66.7
CD45RA-
33.2
CD45RA
CCR7
Q1
5.14
N
Q2
33.1
TEMRA
61.0
Q3
0.79
CD27
CCR7
Q1
TM
43.0
CM
Q2
9.40
Q4
46.8
EM
Q3
0.79
CD27
CCR7

**C**

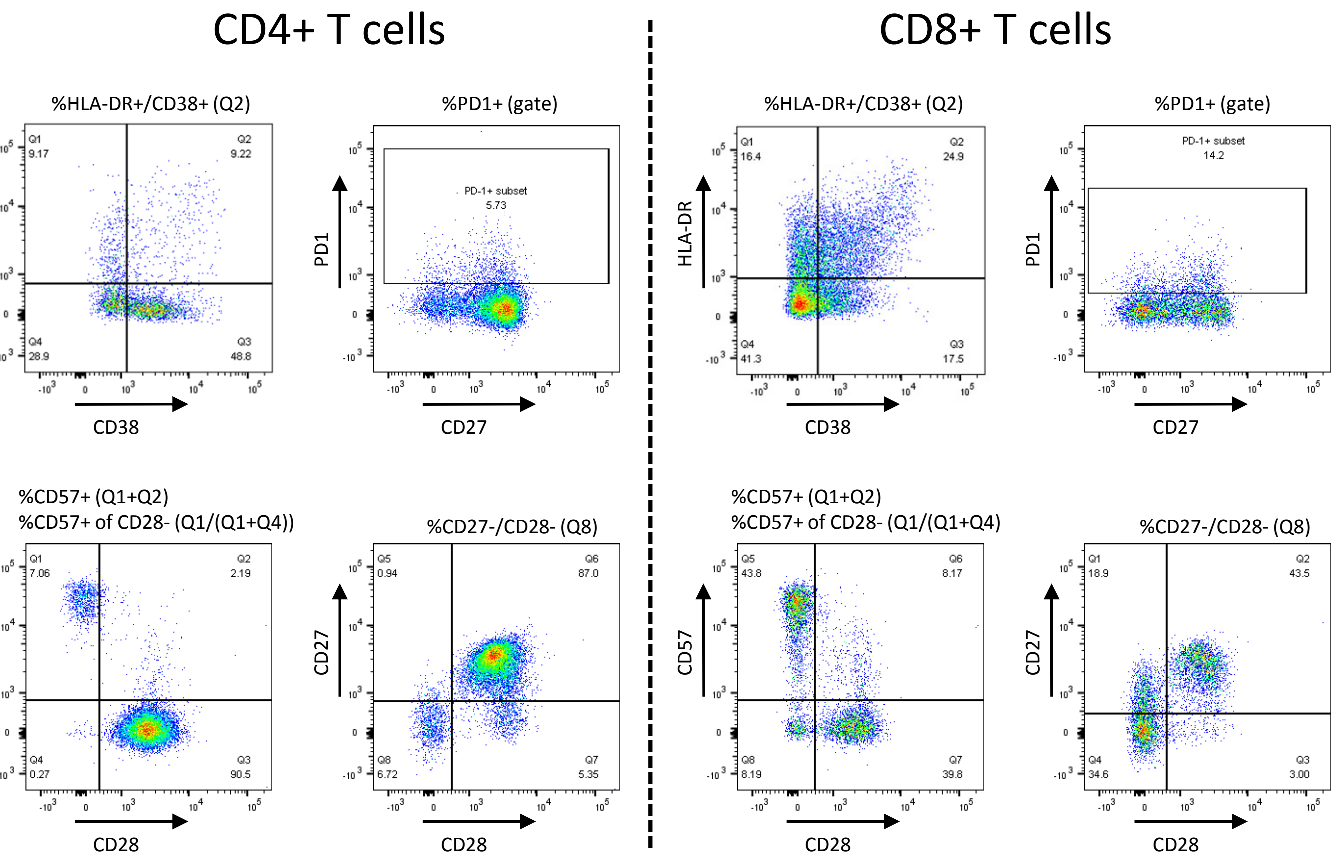


**S1 Fig. Gating strategy flow cytometry. A.** Gating of CD4+ and CD8+ T cells. **B.** T cell differentiation was defined as the proportion of naïve (N; $CD45RA^{+}CD27^{+}CCR7^{+}$), central memory (CM; $CD45RA^{-}CCR7^{+}CD27^{+}$), transitional memory (TM; $CD45RA^{-}CCR7^{-}CD27^{+}$), effector memory (EM; $CD45RA^{-}CCR7^{-}CD27^{-}$), and terminally differentiated effector memory (TEMRA; $CD45RA^{+}CCR7^{-}CD27^{-}$) within the total CD4 or CD8 T cell population. **C.** T cell activation was defined as the proportion of cells that were positive for both CD38 and HLA-DR within the total CD4 or CD8 T cell population. T cell exhaustion was defined as the proportion of PD1 positive cells within the total CD4 or CD8 T cell population. Terminally differentiated T cells were defined as proportion of CD57 positive cells within the total CD4 or CD8 T cells population, the proportion of cells negative for both CD27 and CD28 within the total CD4 or CD8 T-cells population, or the proportion of CD57 positive within the $CD28^{-}CD4^{+}$ or $CD28^{-}CD8^{+}$ T cell population.
